# Targeting ATF6 reduces pathological neovascularization and improves visual outcomes in retinal disease models

**DOI:** 10.1038/s41598-025-15393-y

**Published:** 2025-09-26

**Authors:** Allyssa Bradley, Soyoung Park, Soyeon Park, Kyle Kim, Angela Galdamez, Hyejung Min, Monica Sophia Diaz-Aguilar, M. Elizabeth Hartnett, Eun-Jin Lee, Jonathan H. Lin

**Affiliations:** 1 Department of Pathology, School of Medicine, Stanford University, Palo Alto, CA USA; 2 Department of Ophthalmology, School of Medicine, Stanford University, Palo Alto, CA USA; 3 VA Palo Alto Health Care System, Department of Veterans Affairs, Veterans Health Administration, Palo Alto, CA United States; 4 Rush Medical College, Rush University, Chicago, IL USA; 5 School of Medicine, Stanford University, 300 Pasteur Dr. L235, Palo Alto, CA 94305 USA

**Keywords:** Retina, Angiogenesis, Retinopathy of prematurity, Retinal diseases

## Abstract

Pathological retinal neovascularization is a cause of vision loss in diseases including retinopathy of prematurity (ROP), wet age-related macular degeneration (AMD), and diabetic retinopathy. The Unfolded Protein Response (UPR) is an intracellular signal transduction mechanism that is activated by ER stress and upregulates many proteins, including angiogenesis factors like VEGF and HIF-1α. This suggests that UPR genes and pathways may drive retinal angiogenesis. Here, we tested the role of the UPR regulator Activating Transcription Factor 6 (ATF6) in pathological and developmental retinal angiogenesis. We induced pathological retinal neovascularization in *Atf6*^*−/−*^ mice using the oxygen-induced retinopathy (OIR) model and found significantly preserved visual function, accompanied by decreased retinal neovascularization, endothelial cell proliferation, and UPR transcriptional program induction. When we chemically blocked ATF6 signaling by intraocular injection of the small molecule Ceapin-A7, we also saw suppressed retinal expression of UPR genes. Additionally, in postnatal day 7 *Atf6*^−/−^ mice when the retinal vasculature is developing in response to physiologic intraocular hypoxia, there was a transient but significant defect in pruning and retinal blood vessel extension. Together, our results demonstrate ATF6’s causal role in developmental and pathological retinal angiogenesis and highlight its potential as a therapeutic target to preserve vision in retinal neovascularization diseases.

**Supplementary Information:**

The online version contains supplementary material available at 10.1038/s41598-025-15393-y.

## Introduction

The retinal vasculature begins developing at about 14–16 weeks of gestation in humans and shortly after birth in mice. Initially, the developing eye is nourished by the hyaloid vasculature, a transient network of vitreous vessels that regresses as the retinal vasculature forms. Retinal blood vessels first sprout from the optic nerve head and grow radially toward the periphery, guided by hypoxia-triggered angiogenic signals from avascular areas^1–7^. Disruptions in these signals can impair blood vessel formation, as seen in premature infants receiving high supplemental oxygen therapy, which can slow the progression of angiogenesis and is one of the factors involved in the pathophysiology of the blinding disease retinopathy of prematurity (ROP)^8–10^. In severe cases, ROP progresses to a more advanced stage, marked by abnormal blood vessel growth into the vitreous, along with greater vessel tortuosity and dilation^11–13^. This retinal neovascularization can be experimentally modeled in mice and rats due to the postnatal development of their retinal vasculature. Mice offer the opportunity to study molecular mechanisms more easily than rats. In the mouse oxygen-induced retinopathy (OIR) model, postnatal mice are exposed to hyperoxia and then returned to ambient oxygen levels to induce “relative hypoxia” and retinal neovascularization^5,14^.

A growing body of evidence implicates Unfolded Protein Response (UPR) signaling as a molecular mechanism driving neovascularization^15–19^. Hypoxia, a potent inducer of neovascularization, activates UPR pathways regulated by Inositol-Requiring Enzyme 1 (IRE1), Protein Kinase R (PKR)-like Endoplasmic Reticulum Kinase (PERK), and Activating Transcription Factor 6 (ATF6) through oxidative protein misfolding and subsequent ER stress^20–28^. These pathways enhance cellular survival under stress by upregulating chaperones, protein-folding enzymes, and degradation cofactors for misfolded proteins. Additionally, UPR activation induces critical angiogenesis regulators such as Vascular Endothelial Growth Factor (VEGF)^29,30^. ER stress exacerbates pathological ocular neovascularization in mice^16,31,32^. ATF6 directly modulates VEGF gene expression and stabilizes the VEGF protein^23,29^ and small molecule ATF6 activation promotes blood vessel development in vitro^33^. Inhibition of ER stress pathways, including ATF6, has been reported to reduce retinal neovascularization^23,34^. To expand on these findings and assess if ATF6 impacted visual outcomes, we investigated retinal vascular development under physiological hypoxia and pathological neovascularization in *Atf6*^−/−^ mice.

*Atf6*^*−/−*^ mice do not produce the Atf6 protein due to a premature stop codon in exon IV but remain viable in a standard laboratory vivarium, making them a valuable model for studying ATF6 function in vivo^35,36^. These mice develop sensorineural hearing loss by 2 months of age^37^. *Atf6*^*−/−*^ mice have no defects in retinal vasculature, lamination, and function in studies from P15 through ~ 1 year, but afterward, they exhibit mild late-onset retinal degeneration, which becomes more severe when crossed with the P23H rhodopsin retinitis pigmentosa model^36,38^. *Atf6*^*−/−*^ mouse retinal development (P0-P14) has not been previously evaluated. Beyond the retina, adult *Atf6*^*−/−*^ mice develop liver steatosis and pancreatic beta-cell loss under high-fat diets^39,40^. In the heart, Atf6 deficiency leads to myocardial damage, while in the brain, it increases susceptibility to stroke and neurodegenerative diseases^41,42^. In some cases, Atf6 loss has beneficial effects, such as resistance to paralysis in multiple sclerosis models^43^. These prior studies indicate that loss of Atf6 may cause positive or negative effects in vivo, depending on cell and tissue type.

In this study, we investigate ATF6’s role specifically in retinal blood vessel development in a pathological retinal neovascularization OIR model and during normal developmental physiological intraocular hypoxia.

## Results

### Preserved retinal function and reduced neovascularization after OIR in *Atf6*^−/−^ mice

**Fig. 1:**
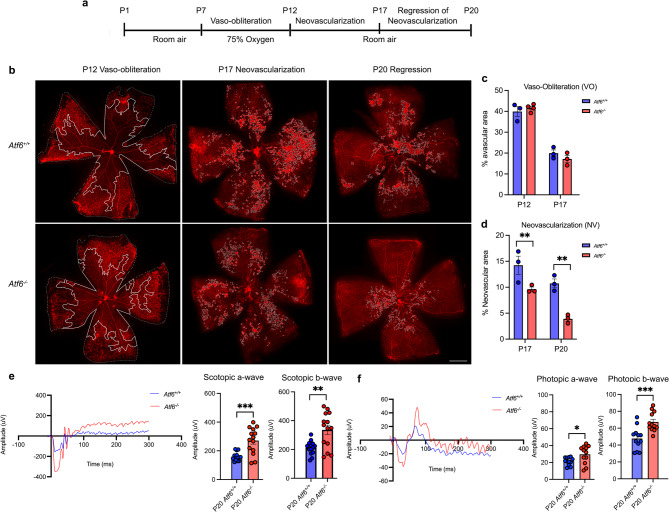
Atf6 deficiency enhances visual function and reduces neovascularization in OIR.** (a)** Timeline of the mouse OIR model. Neonatal mice and their nursing mother were kept in room air from birth through postnatal (P) 7. In the vaso-obliteration phase, P7 mice were transferred from room air into 75% oxygen until P12, then returned to room air. During the neovascularization phase, blood vessels developed pathologic retinal neovascularization, which peaked at P17 and subsequently regressed. **(b)** Wholemount retinas stained for endothelial cells with IB4 (red) in *Atf6*^*+/+*^ and *Atf6*^*−/−*^ OIR mice at P12 (avascular area highlighted with solid white line; vascular area highlighted with white-dotted lines) and at P17 and P20 (neovascular tufts highlighted with white-dotted lines). **(c)** The percentage of avascular area at P12 and P17 showed no significant difference between *Atf6*^*+/+*^ and *Atf6*^*−/−*^ mice (each point represents one retina per mouse, data represents mean ± SEM, *p* > 0.05, *two-way ANOVA*, *n* = 3 or 4/group). **(d)** The percentage of neovascular area at P17 and P20 was significantly reduced in *Atf6*^*−/−*^ mice compared to *Atf6*^*+/+*^ mice (each point represents one retina per mouse, data represents mean ± SEM, **p < 0.01, *two-way ANOVA*, *n* = 3/group). **(e)** Representative ERG waveforms generated by scotopic intensity (40 (S) cd.s/m^2^) for P20 *Atf6*^*+/+*^ (blue line) and *Atf6*^*−/−*^ (red line) retinas (left). Scotopic ERG responses showed significantly greater a- and b-wave amplitudes in *Atf6*^−/−^ mice compared to *Atf6*^+/+^ controls (data represents mean ± SEM, **p ≤ 0.01, ***p ≤ 0.001; *Welch t-test*, *n* = 7/group). **(f)** Representative ERG waveform generated by photopic intensity (10 (S) cd.s/m^2^) for P20 *Atf6*^*+/+*^ (blue line) and *Atf6*^*−/−*^ (red line) retinas (left). Photopic a- and b-wave amplitudes were significantly higher in *Atf6*^*−/−*^ mice compared to *Atf6*^*+/+*^ mice (data represents mean ± SEM, *p ≤ 0.05, ***p ≤ 0.001, *Welch t-test*, *n* = 6/group). Scale bar, 500 μm.

To investigate the role of Atf6 in pathological retinal angiogenesis, we subjected *Atf6*^*−/−*^ mice to the oxygen-induced retinopathy (OIR) paradigm. First, both *Atf6*^*+/+*^ and *Atf6*^*−/−*^ mice were placed in 75% oxygen (hyperoxia) from P7 to P12 (Fig. 1a). This hyperoxic exposure during retinal vasculature development induced vaso-obliteration indistinguishably in both *Atf6*^*+/+*^ and *Atf6*^*−/−*^ retinas (Fig. 1b, left panels). When mice were returned to room air (relative hypoxia), reparative and pathological angiogenesis occurred, and neovascular growth peaked at P17 (Fig. 1b, center panels). By P20, neovascular regression was observed^5,14,44^ (Fig. 1b, right panels). To assess the impact of Atf6 loss on vascular growth, we quantified the percentage of avascular area at P12, P17, and P20, and neovascular area at P17 and P20. Vaso-obliteration did not significantly differ between *Atf6*^+/+^ and *Atf6*^−/−^ mice at P12 (*p* = 0.87) or P17 (*p* = 0.66) (Fig. 1c). Both *Atf6*^+/+^ and *Atf6*^−/−^ OIR mice showed a significant and similar reduction in vaso-obliteration between P12 and P17 (****p < 0.0001 for both *Atf6*^*+/+*^ and *Atf6*^*−/−*^ retinas). P20 showed minimal avascular zones, less than 10%, consistently with previous studies (Supplemental Fig. 1)^45,46^. In contrast, neovascularization was significantly reduced in *Atf6*^−/−^ mice compared to *Atf6*^+/+^ mice at both P17 (**p < 0.01) and P20 (**p < 0.01). Additionally, neovascularization decreased significantly from P17 to P20 in both *Atf6*^+/+^ (*p = 0.014) and *Atf6*^−/−^ mice (**p < 0.01, *two-way ANOVA*, Fig. 1d).

To evaluate whether the sample sizes used for each experiment were adequate to detect biologically meaningful differences, we performed post hoc power analyses using GraphPad Prism (version 10). We calculated group means, 95% confidence intervals (CIs), and effect sizes (Cohen’s d). At P12, the mean avascular area was slightly lower in *Atf6*^+/+^ retinas (39.92%; 95% CI: 29.62–50.22) than in *Atf6*^−/−^ retinas (41.60%; 95% CI: 38.77–44.43), with a small effect size (Cohen’s d ≈ 0.53), indicating no biologically meaningful difference. At P17, a modest reduction in avascular area was observed in *Atf6*^−/−^ retinas (17.18%; 95% CI: 9.69–24.67) compared to *Atf6*^+/+^ retinas (19.94%; 95% CI: 13.27–26.61), with a moderate effect size (Cohen’s d ≈ 0.97), indicating no statistically significant difference between groups.

In contrast, *Atf6*^−/−^ retinas exhibited a significant and biologically meaningful reduction in neovascularization. At P17, the neovascular area was 9.63% (95% CI: 7.85–11.41) in *Atf6*^−/−^ retinas versus 14.24% (95% CI: 6.67–21.80) in *Atf6*^*+/+*^ controls, with a large effect size (Cohen’s d ≈ 2.08). At P20, neovascularization was further reduced in *Atf6*^−/−^ retinas (3.91%; 95% CI: 1.83–5.99) relative to *Atf6*^*+/+*^ controls (10.74%; 95% CI: 7.03–14.45), with a very large effect size (Cohen’s d ≈ 5.64). Post hoc power analysis indicated high statistical power (~ 99.8%), confirming that the sample size was sufficient to detect this robust biological effect. These results indicate that Atf6 deficiency is associated with a reproducible and statistically supported reduction in pathological neovascularization, supported by statistical and effect size analyses.

To assess visual function in the absence of Atf6 in OIR mouse retinas, we performed electroretinograms (ERGs) at P20. Waveforms of the scotopic and photopic ERG responses were collected from both *Atf6*^+/+^ and *Atf6*^−/−^ OIR retinas. Both scotopic (Fig. 1e) and photopic (Fig. 1f) ERG responses showed significantly greater a- and b-wave amplitudes in *Atf6*^−/−^ mice compared to *Atf6*^+/+^ controls (*p ≤ 0.05, **p ≤ 0.01, ***p ≤ 0.001; Welch’s t-test). Together, these histologic and functional imaging studies reveal that *Atf6*^*−/−*^ mice are resistant to neovascularization and retain better visual function compared to control *Atf6*^+/+^ mice after OIR, indicating that the inhibition of Atf6 function protects against OIR-induced pathological angiogenesis and subsequent vision loss.

### Reduced endothelial cell proliferation in *Atf6*^−/−^ OIR model

**Fig. 2:**
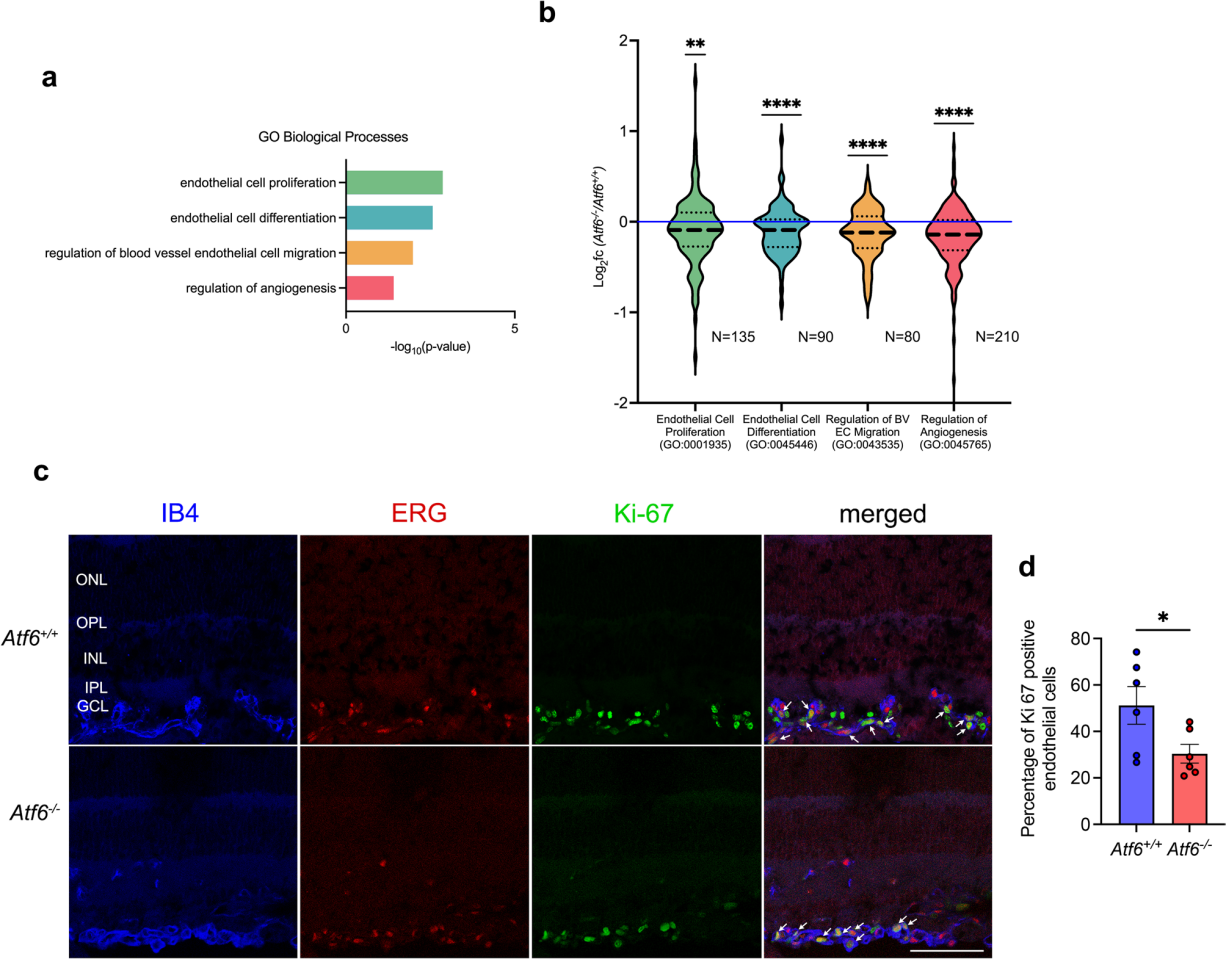
Endothelial cell proliferation is reduced in *Atf6*^−/−^ OIR mice during retinal neovascularization. **(a)** RNA sequencing was performed on P17 (the peak of retinal neovascularization) *Atf6*^*+/+*^ and *Atf6*^*−/−*^ OIR mouse retinas. Functional enrichment analysis using the Gene Ontology (GO) Biological Processes database via g:Profiler on all significantly differentially expressed genes (2,096 genes) revealed significant changes in terms for endothelial cell proliferation, endothelial cell differentiation, regulation of blood vessel endothelial cell migration, and regulation of angiogenesis (*n* = 5/group). The x-axis shows the -log_10_(p-value) for each term. **(b)** Violin plots show gene set expression levels for the terms identified in functional enrichment analysis (GO:0001935 endothelial cell proliferation, green; GO:0045446 endothelial cell differentiation, blue; GO:0043535 regulation of blood vessel (BV) endothelial cell (EC) migration, yellow; GO:0045765 regulation of angiogenesis, red) were all significantly downregulated in P17 *Atf6*^*−/−*^ retina transcriptomes, expressed as log_2_(fold change) compared to P17 *Atf6*^*+/+*^ retinas. The thick dashed line indicates the median log_2_(fold change), the thin dashed lines demarcate the top and bottom quartiles, and the blue line indicates 1x fold change (**p ≤ 0.01, ****p ≤ 0.0001, *Two-Tailed Wilcoxon Signed Rank Test*, *n* = 5/group). The complete gene sets are shown in Supplemental Table 3. **(c)** Representative images of vertical retinal sections labeled with IB4 (blue), ERG (red), and Ki-67 (green) from P17 *Atf6*^*+/+*^ and *Atf6*^*−/−*^ retinas (*n* = 3/group). In the merged image, colocalization of ERG and Ki-67 inside of IB4 areas (arrows) indicate proliferating inner retina blood vessel endothelial cells. **(d)** Percentage of Ki-67-immunoreative blood vessel endothelial cells was significantly lower in *Atf6*^*−/−*^ retinas compared to *Atf6*^*+/+*^ retinas (data represent mean ± SEM, *p ≤ 0.05, *Student’s t-test*, *n* = 3/group). *ONL* outer nuclear layer; *OPL* outer plexiform layer; *INL* inner nuclear layer; *IPL* inner plexiform layer; *GCL* ganglion cell layer. Scale bar = 50 μm.

To understand the molecular mechanisms underlying the reduction in neovascularization in *Atf6*^*−/−*^ OIR mice, we compared bulk RNA sequencing (RNA-Seq) on retinas collected from P17 *Atf6*^+/+^ and *Atf6*^−/−^ OIR mice (*n* = 5/group). Retinas were collected at P17, the peak of neovascular response, consistent with previous OIR studies^5,14,44^. Out of 15,524 total genes identified by RNA-Seq, 2,096 (identified by DESeq2 with *p* ≤ 0.05 and FPKM > 0.1, out of 15,524 transcripts) were significantly differentially expressed in *Atf6*^−/−^ mice compared to controls by DESeq2 analysis (Supplemental Table 1). These differentially expressed genes (DEGs) were uploaded to g:Profiler (https://biit.cs.ut.ee/gprofiler*)* for functional enrichment analysis using the Gene Ontology (GO) Biological Processes database to identify significantly altered pathways (Supplemental Table 2). Our analysis revealed significant enrichment of pathways associated with blood vessel growth including endothelial cell (EC) proliferation, EC differentiation, regulation of blood vessel EC migration, and regulation of angiogenesis (Fig. 2a). Endothelial cell proliferation is a hallmark of proliferative retinopathy, essential for both regenerative and pathological angiogenesis^47–49^. To further examine these angiogenic processes in *Atf6*^−/−^ OIR retinas, we analyzed expression of the constituent genes within the significantly-enriched angiogenesis-associated GO terms: EC proliferation (GO:0001935, green, *n* = 135 genes), EC differentiation (GO:0045446, blue, *n* = 90 genes), regulation of blood vessel EC migration (GO:0043535, yellow, *n* = 80 genes), and regulation of angiogenesis (GO:0045765, red, *n* = 210 genes) (Fig. 2b, Supplemental Table 3). Compared to control retinas, *Atf6*^*−/−*^ retinas exhibited a significant reduction in the mean expression of all four gene sets: **p < 0.01 for EC proliferation and ****p < 0.0001 for the other three categories, based on a Two-Tailed Wilcoxon Signed-Rank Test (*n* = 5/group, Fig. 2b). These findings are illustrated in the violin plots of Fig. 2b, where green indicates **p ≤ 0.01 and blue, yellow, and red indicate ****p ≤ 0.0001. These findings support that genes associated with endothelial cell function including proliferation, differentiation, migration, and angiogenesis are significantly downregulated in *Atf6*^*−/−*^ OIR retinas. Furthermore, these transcriptomic findings align with our histologic findings that retinal neovascularization was reduced in *Atf6*^*−/−*^ mice.

Previous studies have shown that OIR mouse retinas typically exhibit an increase in endothelial cells, heightened cell proliferation, and enhanced vaso-proliferation, particularly during the neovascular phase^47,50^. To further investigate whether the reduction of neovascularization in *Atf6*^*−/−*^ OIR retinas was due to a lower number of proliferating endothelial cells in neovascular tufts, we performed triple labeling using the endothelial marker IB4, the endothelial cell nuclei marker ERG, and the proliferation marker Ki-67 (Fig. 2c). In *Atf6*^+/+^ OIR retinas, numerous Ki-67-positive endothelial cells were observed, co-localized with ERG in IB4-positive blood vessels (Fig. 2c, top panels). In contrast, *Atf6*^*−/−*^ OIR retinas exhibited a marked reduction in the number of Ki-67-positive endothelial cells, indicating decreased endothelial cell proliferation (Fig. 2c, bottom panels). The merged images confirm a significant reduction in proliferating endothelial cells (IB4^+^, ERG^+^, and Ki-67^+^) in *Atf6*^*−/−*^ retinas compared to *Atf6*^+/+^ retinas (*p ≤ 0.05, *Student’s t-test*; Fig. 2d). These RNA-Seq and histologic data demonstrate that inhibition of Atf6 reduces endothelial cell proliferation in the OIR mouse retina.

### Phototransduction genes upregulated in *Atf6*^−/−^ OIR mice with unchanged ONL thickness

**Fig. 3:**
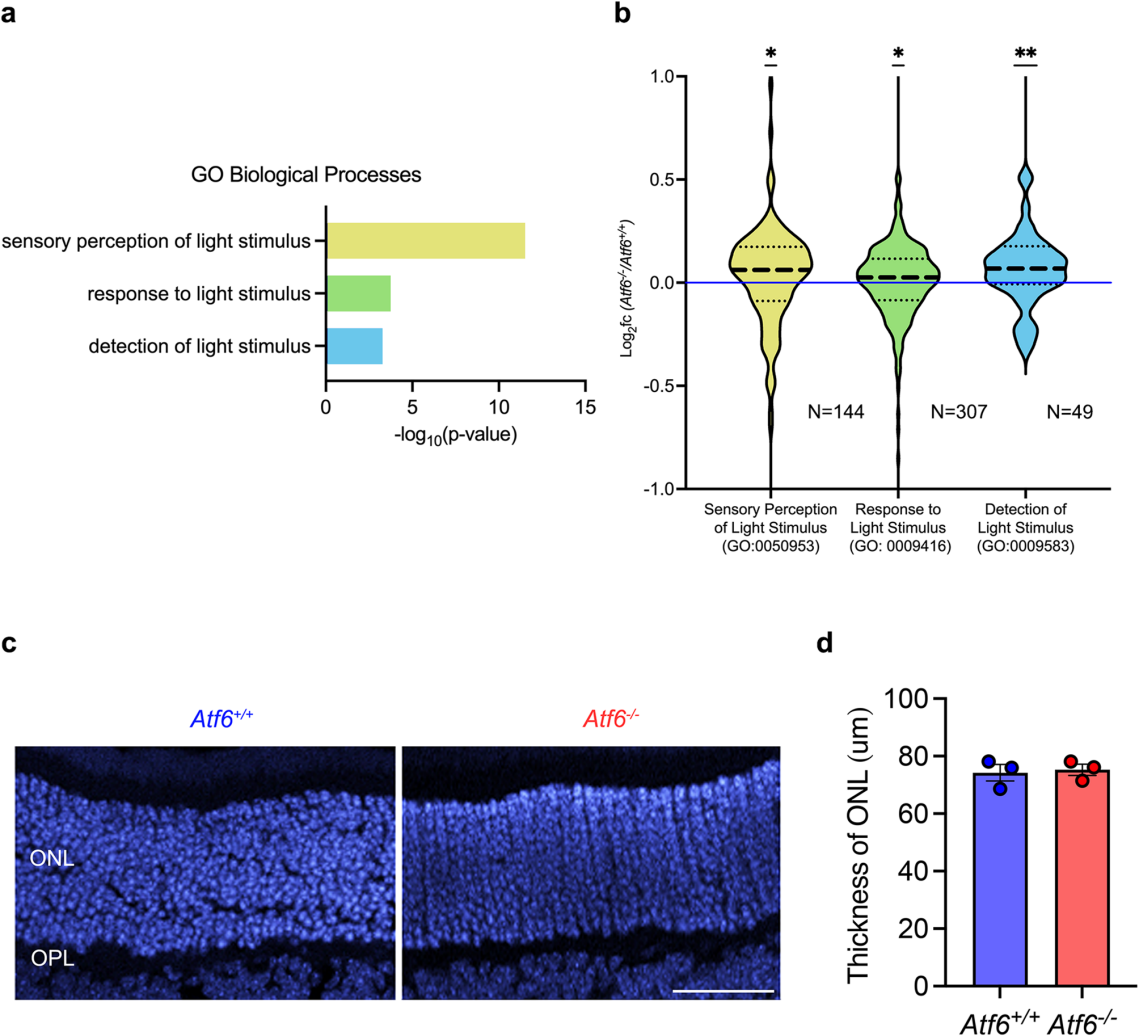
Phototransduction genes upregulated in *Atf6*^−/−^ retinas with no change in retinal ONL in OIR.** (a)** RNA sequencing was performed on P17 (the peak of retinal neovascularization) *Atf6*^*+/+*^ and *Atf6*^*−/−*^ OIR mouse retinas. Functional enrichment analysis using the Gene Ontology (GO) Biological Processes database via g:Profiler on all significantly differentially expressed genes (2,096 genes) revealed significant changes in terms for sensory perception of light stimulus, response to light stimulus, and detection of light stimulus (*n* = 5/group). The x-axis shows the -log_10_(p-value) for each term. **(b)** Violin plots show gene set expression levels for the terms identified in functional enrichment analysis (GO:0050953 sensory perception of light stimulus, yellow; GO:0009416 response to light stimulus, green; and GO:0009583 detection of light stimulus, blue) in P17 *Atf6*^*−/−*^ OIR retina RNA-Seq transcriptomes, expressed as log_2_(fold change) compared to P17 *Atf6*^*+/+*^ OIR retinas (*p ≤ 0.05, **p ≤ 0.01, *Two-Tailed Wilcoxon Signed Rank Test*, *n* = 5/group). The complete gene sets are shown in Supplemental Table 4. **(c)** Representative images of the retinal outer nuclear layer (ONL) approximately 500 μm away from the optic disc in P17 *Atf6*^*+/+*^ and *Atf6*^*−/−*^ retinas, labeled with DAPI (*n* = 3/group). **(d)** Thickness of ONL was not significantly different between *Atf6*^*+/+*^ and *Atf6*^*−/−*^ retinas (data represent mean ± SEM, *p* > 0.05, *Student’s t-test*, *n* = 3/group). *ONL* outer nuclear layer; *OPL* outer plexiform layer. Scale bar = 50 μm.

We found that Atf6 deficiency improved visual function in P20 OIR mice, as evidenced by enhanced ERG responses (Fig. 1e, f). GO comparison of the retinal transcriptome between *Atf6*^*−/−*^ and control mice revealed significant enrichment of terms related to visual function (Supplemental Table 2), including sensory perception of light stimulus (GO:0050953, yellow, *n* = 144), response to light stimulus (GO:0009416, green, *n* = 307), and detection of light stimulus (GO:0009583, blue, *n* = 49; Fig. 3a). To investigate the molecular basis of this improvement, we analyzed the expression levels of the gene sets corresponding to these GO terms (Supplemental Table 4). Compared to *Atf6*^+/+^ retinas, *Atf6*^*−/−*^ retinas exhibited significantly higher mean expression across all three gene sets: *p ≤ 0.05 for sensory perception of light stimulus and response to light stimulus, and **p ≤ 0.01 for detection of light stimulus, based on a Two-Tailed Wilcoxon Signed-Rank Test (*n* = 5/group). These differences are illustrated in the violin plots in Fig. 3b, where yellow and green indicate *p ≤ 0.05 and blue indicates **p ≤ 0.01. These findings support that genes associated with visual function including sensory perception of light stimulus, response to light stimulus, and detection of light stimulus are significantly upregulated in *Atf6*^*−/−*^ OIR retinas. Furthermore, Gene Set Enrichment Analysis (GSEA) confirmed marked enrichment of the sensory perception of light stimulus gene set (FDR = 0.01) in the *Atf6*^−/−^ OIR retina transcriptome (Supplemental Fig. 2). Together, these transcriptomic results suggest that the protective effect of Atf6 deficiency against neovascularization is accompanied by preservation of visual response pathways, consistent with the improved ERG responses observed in *Atf6*^−/−^ retinas (Fig. 1).

Lastly, to determine whether the thickness of the retinal outer nuclear layer (ONL) differed between *Atf6*^*+/+*^ and *Atf6*^*−/−*^ OIR retinas, we measured the ONL thickness in vertical cross-sections (Fig. 3c). No significant difference in ONL thickness was observed between *Atf6*^*−/−*^ (data represent mean ± SEM, 75.2 ± 1.9 μm) and *Atf6*^*+/+*^ retinas (74.2 ± 2.8 μm, *p =* 0.6, *Student’s t-test*, *n* = 3; Fig. 3d). This indicates that the enhanced visual response and increased gene expression in *Atf6*^*−/−*^ mice are not due to differences in thickness of ONL.

### Downregulation of UPR genes after genetic or chemical ATF6 inhibition

**Fig. 4:**
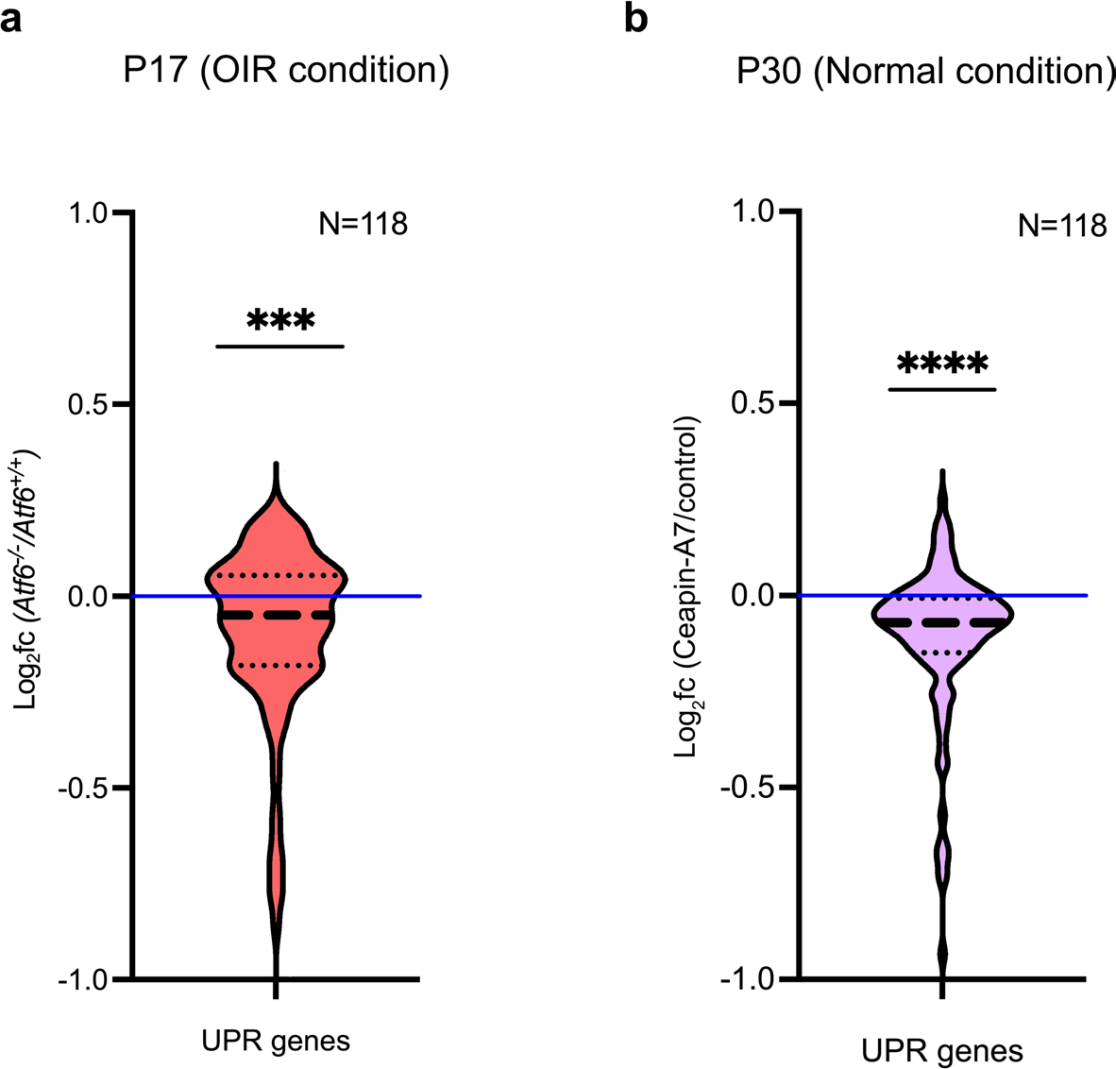
Genetic or chemical Atf6 inactivation downregulated UPR-regulated gene set. **(a)** Violin plot shows expression levels of 118 UPR-regulated genes in P17 *Atf6*^*−/−*^ OIR retina RNA-Seq transcriptomes. Expression levels are shown as log_2_fold change compared to *Atf6*^*+/+*^ OIR retinas (red violin plot, ***p ≤ 0.001, *Two-Tailed Wilcoxon Signed Rank Test*, *n* = 5/group). The complete UPR gene set is shown in Supplemental Table 5. **(b)** Violin plot shows expression levels of 118 UPR-regulated genes in wild-type P30 retinas treated with Ceapin-A7, 24 h post-injection. The thick dashed line indicates the median log_2_(fold change), the thin dotted lines indicate the top and bottom quartiles, and the blue line indicates 1x fold change (purple violin plot, ****p ≤ 0.0001, *Two-Tailed Wilcoxon Signed Rank Test*, *n* = 5/group; Supplemental Table 7).

ATF6 is a key component of the UPR pathway, which is activated in response to ER stress. Previous studies have shown that ER stress is significantly elevated in OIR retinas, with activation of UPR signaling molecules such as GRP78, PERK, and eIF2A, some of which are also linked to pathological neovascularization^32,34,51^. Since ATF6 and other UPR pathways upregulate many genes aiding ER stress adaptation^16,23,33 ^we investigated how UPR-regulated gene expression was altered in the transcriptomic data collected from our *Atf6*^−/−^ OIR retinas. Using RNA-Seq data, we examined the expression of a previously published panel of 118 genes transcriptionally regulated by the IRE1/XBP1, ATF6, and PERK/ATF4 UPR pathways^35,52–60^ in P17 *Atf6*^*+/+*^ and *Atf6*^*−/−*^ OIR mouse retinas (Supplemental Table 5). The 118-gene UPR gene panel was significantly downregulated in *Atf6*^*−/−*^ versus *Atf6*^*+/+*^ retinas, as shown in the violin plot (red violin plot, ***p ≤ 0.001, *Two-Tailed Wilcoxon Signed Rank Test*, *n* = 5/group; Fig. 4a). These findings reveal that the UPR transcriptional program is downregulated in *Atf6*^−/−^ mice compared to *Atf6*^+/+^ after OIR.

To further test if UPR gene expression was disrupted by loss of Atf6, we chemically inhibited Atf6 using the small molecule Ceapin-A7^61^. Intravitreal injections of Ceapin-A7 were performed on P30 *Atf6*^+/+^ mice, and bulk RNA-Seq transcriptomes were analyzed from saline-treated and Ceapin-A7-treated retinas 24 h post-injection. This design allowed us to isolate direct molecular effects of Atf6 inhibition without the confounding variables present in the OIR model, such as fluctuating oxygen levels and active neovascular remodeling. We identified 15,417 (expression > 0.1 FPKM) transcripts expressed in Ceapin-A7-treated retinas (Supplemental Table 6). Similarly to our findings in the *Atf6*^−/−^ mice retinas collected after OIR, the expression levels of the UPR genes (*n* = 118, Supplemental Table 7) were significantly downregulated in Ceapin-A7-treated versus saline-treated retinas (purple violin plot, ****p ≤ 0.0001, *Two-Tailed Wilcoxon Signed Rank Test*, *n* = 5/group; Fig. 4b). Our results indicate that either genetic or chemical inhibition of Atf6 led to a downregulation of UPR genes relative to controls, in OIR and in normal conditions. These findings support that the reduced retinal neovascularization observed in *Atf6*^−/−^ mice under OIR conditions is caused by a diminished UPR transcriptional response.

### Early retinal angiogenesis is delayed in *Atf6*^−/−^ mice

During early retinal development in mice (P0-P7), physiologic hypoxia in the eye triggers the formation of new retinal blood vessels, emerging from the optic nerve head and extending from the central to the peripheral retina^4,6^. As these new vessels form and alleviate hypoxia, vascular growth continues radially toward the retinal periphery^7^. Since Atf6 deficiency suppressed neovascularization in the pathologic OIR model, we investigated if Atf6 loss also affects physiologic hypoxia-driven retinal vascular development during this early postnatal period. To address this, we analyzed retinal vasculature in *Atf6*^*+/+*^ and *Atf6*^*−/−*^ mice at P3, P7, P10, and P14. These time points were selected to capture key phases of retinal angiogenesis^62^. At P3, the superficial vascular network begins to form as a dense, immature plexus with active sprouting at the leading edge. By P7, the superficial network is expanding toward the peripheral retina and vertical sprouting from superficial veins into the deep retinal layers begins to initiate, marking the onset of vascular stratification. P10 represents a phase of active vertical sprouting and further elaboration of the intermediate and deep vascular layers, while capillary sprouting and remodeling continue within the superficial plexus. By P14, the vascular network reaches near-maximum complexity, with established layers and ongoing remodeling. These four time points represent a continuum from initial plexus formation (P3) through onset and regulation of stratification (P7-P10) to late-stage vascular maturation (P14)^62^. This developmental timeline provides a framework to assess how Atf6 influences each stage of retinal angiogenesis.

**Fig. 5:**
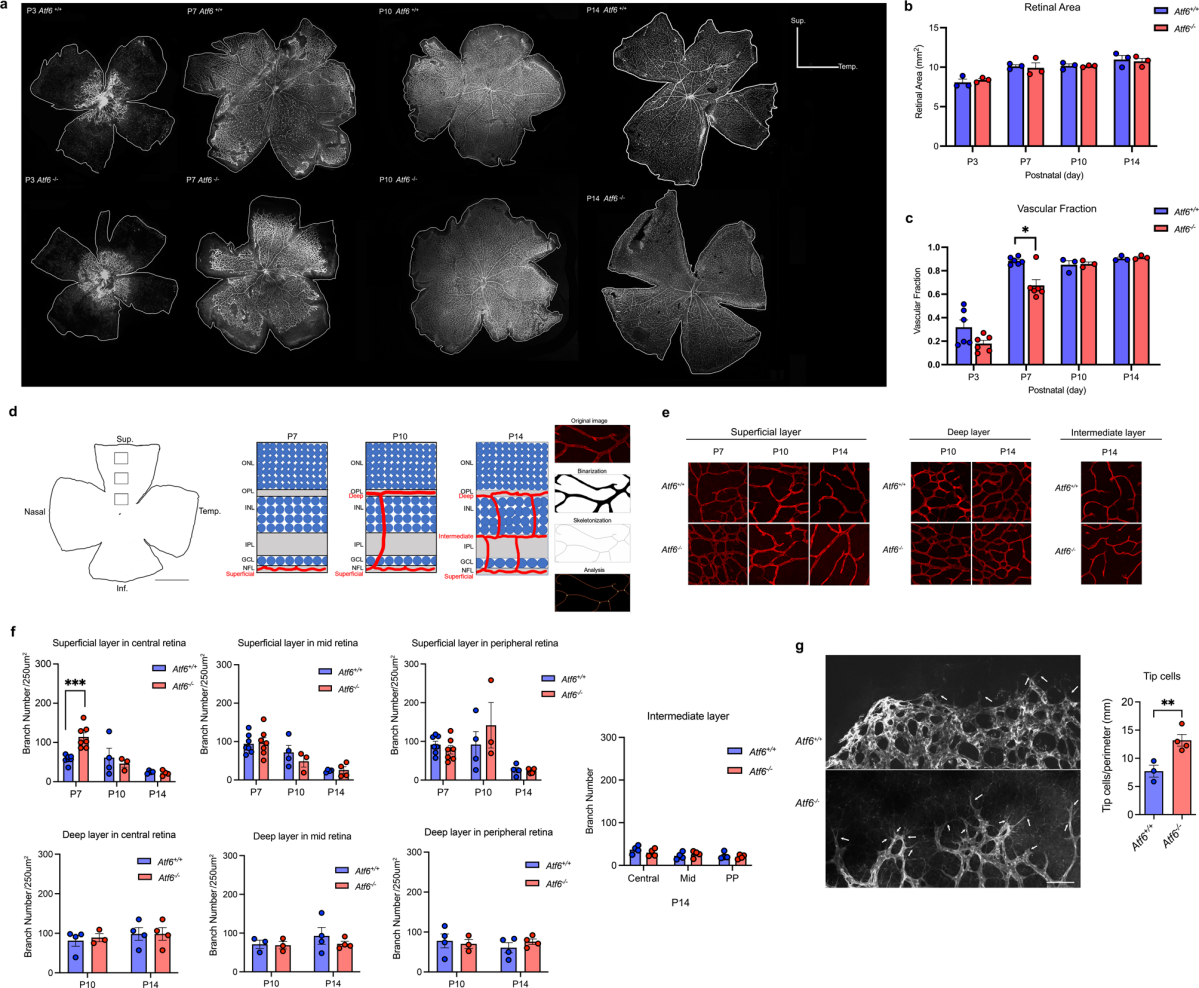
Early retinal angiogenesis is delayed in *Atf6*^−/−^ mice. **(a**) Wholemounts of *Atf6*^*+/+*^ and *Atf6*^*−/−*^ mice at P3, P7, P10, and P14 labeled with IB4 to visualize vasculature. Retina area is outlined. **(b)** Retina area did not differ significantly between *Atf6*^*+/+*^ and *Atf6*^*−/−*^ retinas at P3, P7, P10, and P14 (data represent mean ± SEM, *p* > 0.05, *two-way ANOVA*, *n* = 3/group). **(c)** Vascular fraction was significantly reduced in *Atf6*^*−/−*^ retinas compared to *Atf6*^*+/+*^ retinas at P7 (*n* = 6/group) but showed no significant difference at P3 (*n* = 6/group) or P10 and P14 (*n* = 3/group) (data represent mean ± SEM, *p < 0.05, *two-way ANOVA*). **(d)** Wholemounts were analyzed for branch number in fields from central (250 μm away from the optic disc), mid-peripheral (750 μm away from the optic disc), and peripheral (1.25 mm away from the optic disc) regions (black boxes, 250 μm x 250 μm) in the superior region (left). Schematic of vascular plexus development (center). ImageJ (FIJI) quantification workflow (right). **(e)** Confocal microscopy images of IB4-stained wholemounts at P7 (superficial layer), P10 (superficial and deep layers), and P14 (superficial, deep, and intermediate layer) in *Atf6*^*+/+*^ and *Atf6*^*−/−*^ retinas. **(f)** Branch counts in the superficial layer at P7, P10, and P14. At P7, branch number is significantly greater in the central retina of *Atf6*^*−/−*^ mice compared to *Atf6*^*+/+*^ mice (data represent mean ± SEM, *p < 0.01, *two-way ANOVA*, *n* = 7/group). At P10 and P14, no differences in the central retina (data represent mean ± SEM, *p* > 0.05, *two-way ANOVA*, *n* = 3–4/group). Mid-peripheral and peripheral regions showed no differences at any time points (P7, *n* = 7/group; P10 and P14, *n* = 3–4/group). Branch numbers in the deep layer at P10 and P14 and the intermediate layer at P14 showed no differences between groups. (**g)** Representative images of endothelial tip cells at P7 in *Atf6*^*+/+*^ and *Atf6*^*−/−*^ retinas labeled with IB4 (left). Tip cell number is significantly greater in *Atf6*^*−/−*^ retinas (right, data represent mean ± SEM, *p < 0.05, *Student’s t-test*, *n* = 3–4/group). *Sup.* superior; *temp.* temporal; *inf.* Inferior. Scale bar = 1 mm (A); 1 mm (D); 50 μm (E); 50 μm (G).

Retinal wholemounts at these representative timepoints were stained with IB4 to visualize vascular growth (Fig. 5a). At P3, blood vessels were visible around the optic nerve head. By P7, these vessels had expanded into a visible network and reached the retinal periphery by P10 in *Atf6*^*+/+*^ retinas. In contrast, *Atf6*^*−/−*^ retinas showed reduced vascular coverage at P7, but this difference resolved by P10, with no significant difference in vascular fraction from controls at P10 and P14. This is consistent with our prior findings that showed no vascular differences in *Atf6*^*−/−*^ mice at older ages^38^. While the total retinal area was similar between *Atf6*^*+/+*^ and *Atf6*^*−/−*^ retinas at all time points (Fig. 5b), the vascular fraction was significantly reduced in *Atf6*^*−/−*^ retinas at P7 (*p < 0.05, *two-way ANOVA*; Fig. 5c), indicating a transient delay in the radial development of superficial vasculature during this period of developmental hypoxia-driven retinal angiogenesis.

Next, we examined the morphology and architecture of the developing blood vessels in more detail at P7 when we found reduced angiogenesis. We quantified vascular branch density in both the superficial and deep retinal layers. Branch density was measured in retinal areas of 250 μm x 250 μm located 250 μm (central), 750 μm (mid-peripheral), and 1250 μm (peripheral) from the optic disc (Fig. 5d). Superficial vasculature was imaged at P7, P10, and P14; deep layer vasculature at P10 and P14; and the intermediate layer at P14, when the plexus becomes more clearly visualized and distinguishable (Fig. 5e). At P7, branch density in the central retina was significantly higher in *Atf6*^*−/−*^ retinas compared to *Atf6*^*+/+*^ retinas, suggesting reduced branch pruning in the absence of Atf6 (***p < 0.001, *two-way ANOVA*; Fig. 5f). In contrast, no significant difference in branch density was observed in the mid-peripheral and peripheral superficial retina between genotypes at P7. By P10, branch pruning had normalized between the two genotypes. In the deep retinal layer, branch density did not differ significantly between genotypes at either P10 or P14 in central, mid-peripheral, or peripheral retina. Similarly, in the intermediate layer, imaged at P14 when it becomes morphologically distinct, branch density remained comparable across all regions between *Atf6*^+/+^ vs. *Atf6*^−/−^ retinas (Fig. 5f). Finally, we quantified tip cells, which are essential for angiogenesis due to their role in VEGF signaling^63–66^. In the *Atf6*^*+/+*^ retinas at P7, the vascular front at the periphery appeared smoother with fewer long filopodia observed along the leading edge. In contrast, *Atf6*^*−/−*^ retinas at P7 exhibited many long filopodia at the leading edge, indicating active sprouting and tip cell formation (Fig. 5g, left). Moreover, the number of tip cells was significantly higher in the *Atf6*^*−/−*^ retinas compared to the *Atf6*^*+/+*^ retinas (**p < 0.01, *Student’s t-test*; Fig. 5g, right). These results identify defects in retinal blood vessel pruning, branching, morphology, and tip cell numbers that underlie a transient delay in retinal angiogenesis seen in *Atf6*^*−/−*^ retinas during early development.

## Discussion

In this study, we explored the role of ATF6 in retinal neovascularization using the OIR model, hypothesizing that ATF6 drives pathological angiogenesis. Hypoxia, a known trigger for neovascularization, activates UPR pathways like IRE1, PERK, and ATF6, which in turn elevate factors like VEGF to promote angiogenesis. Here, we demonstrate that Atf6-deficient mice exhibit reduced blood vessel formation and significantly improved visual function post-hypoxia. Our RNA-Seq analysis showed significant downregulation of angiogenesis-related gene sets in *Atf6*^−/−^ retinas, along with fewer proliferating endothelial cells, suggesting that ATF6 selectively mediates neovascularization.

UPR signaling, such as through ATF6, induces angiogenesis regulators like VEGF, linking ER stress to neovascularization^18,29,30^. Interestingly, VEGF can also increase unfolded proteins in the ER, further activating the UPR^67^. In our study, *Atf6*^−/−^ mice showed resistance to neovascularization post-hypoxia, likely due to decreased endothelial cell proliferation. This reduction was accompanied by downregulation of angiogenesis-related gene sets, including endothelial cell proliferation, endothelial cell differentiation, regulation of blood vessel endothelial cell migration, and regulation of angiogenesis, in the hypoxic retinas of *Atf6*^−/−^ mice. These findings align with prior reports that ATF6 directly regulates VEGF transcription by binding to its promoter and stabilizes VEGF protein to prevent its degradation^23,29^. Additionally, loss of ATF6 may alter oxidative stress responses, potentially affecting ROS-mediated signaling pathways such as HIF-1α stabilization, which is critical for VEGF induction under hypoxia^41^. These findings suggest that ATF6 deficiency not only suppresses VEGF but also disrupts the oxidative stress-mediated angiogenic response in the OIR model. This highlights ATF6’s multifaceted role in the retinal response to oxidative stress and neovascularization, making it an attractive therapeutic target for conditions involving abnormal blood vessel growth.

Our findings identify a role for ATF6 in early retinal vascular development, as evidenced by delayed angiogenesis and reduced pruning in *Atf6*^−/−^ mice at P7. Specifically, we observed delayed outgrowth of the radial superficial vascular plexus and increased branch density in the central retina, suggesting a shift in vessel remodeling dynamics. Pruning in hyperoxic regions is essential for transforming the primitive vascular network into a mature, organized structure^6^. In *Atf6*^+/+^ retinas, the vascular front at P7 appeared smooth, with fewer filopodia, reflecting advanced maturation. In contrast, *Atf6*^−/−^ retinas exhibited increased filopodia, indicative of active sprouting and tip cell formation. Tip cells, driven by VEGF-mediated astrocyte signaling in hypoxic regions, facilitate the radial extension of vasculature to the periphery^63–66^. These observations suggest that ATF6 is important for balancing vascular sprouting and pruning, possibly through modulation of VEGF signaling or other angiogenic pathways. Notably, the developmental delay in angiogenesis observed in *Atf6*^−/−^ mice was transient, with normal vascular formation restored by P10. This suggests the presence of compensatory mechanisms that mitigate the absence of ATF6 during retinal vascular development. One explanation for the initial delay lies in ATF6’s established role in promoting mesoderm formation and differentiation into vascular tissues. Studies have shown that loss-of-function mutations in ATF6 reduce mesodermal marker expression in patient-derived stem cells, indicating a direct influence of ATF6 on early vascular development^33^. However, the restoration of vascularization at later stages is likely due to compensatory mechanisms involving other branches of the UPR. For example, the IRE1 pathway activates stress-response genes that support cell survival and adaptation, and its functional overlap with ATF6 may help offset the loss of ATF6^36,68,69^. IRE1 has also been shown to regulate angiogenic factors, such as VEGF and HIF-1α, by splicing XBP1 mRNA, promoting endothelial cell proliferation and survival under hypoxic conditions^15,23,70,71^. The essential role of IRE1/XBP1 in development is evident in *Xbp-1s*^−/−^ and *Ire1α*^−/−^ mice, which exhibit severe defects and die early in embryogenesis^72,73^. Similarly, *Perk*^−/−^ mice develop skeletal dysplasia at birth, experience postnatal growth retardation, and typically die within four weeks^74,75^. In contrast, adult *Atf6*^−/−^ mice maintain normal retinal vasculature and visual function under standard conditions until late in life^38^. These findings of the various UPR gene knockout mice show that ATF6 ablation causes the least systemic toxicity among the three UPR branches, supporting ATF6 inhibition as a promising, low-toxicity strategy for suppressing pathological neovascularization.

To assess changes in photoreceptor function in *Atf6*^−/−^ mice with OIR, we utilized ERG. Previous studies in rat OIR models^76–78^ and patients with retinopathy of prematurity (ROP)^79,80^ have reported retinal dysfunction, often implicating impaired photoreceptor function due to pathological neovascularization. In our study, *Atf6*^−/−^ OIR mice exhibited increased amplitudes in both scotopic and photopic a- and b-wave ERG responses, suggesting enhanced photoreceptor function compared to *Atf6*^+/+^ OIR mice. The typical reduction in visual response amplitude in OIR is often attributed to dysregulation in photoreceptor outer segment membrane channels, which has been linked to reduced outer segment length^81^. Interestingly, despite the enhanced ERG amplitudes in *Atf6*^−/−^ mice, the outer nuclear layer (ONL) thickness remained comparable between *Atf6*^+/+^ and *Atf6*^−/−^ OIR retinas. This finding aligns with preserved ONL thickness observed in some previous OIR mouse models^81,82^ but retinal thinning has also been observed in some OIR mouse and rat models^80,83–85^. These results suggest that the increase in scotopic and photopic b-wave amplitudes in *Atf6*^−/−^ mice is not due to differences in thickness in ONL but rather reflects functional preservation of the photoreceptors. Gene expression analysis further revealed upregulation of the sensory perception of light stimulus, response to light stimulus, and detection of light stimulus gene sets in *Atf6*^−/−^ OIR retinas. While we did not directly measure photoreceptor outer segment length, the functional data suggest that photoreceptor channels in the outer segment membrane are better preserved in *Atf6*^−/−^ OIR retinas. This preservation may be linked to reduced neovascularization, which alleviates the detrimental effects of pathological angiogenesis on photoreceptor function. These findings highlight a potential protective role of ATF6 inhibition in mitigating angiogenesis-associated photoreceptor dysfunction.

Our findings support that UPR inhibition, demonstrated with *Atf6*^−/−^ mice and the ATF6-specific inhibitor Ceapin-A7, holds promise as a therapeutic strategy for diseases linked to neovascularization. In *Atf6*^−/−^ mice, pathological neovascularization was significantly reduced, accompanied by downregulation of UPR-related genes. Similarly, intravitreal injections of Ceapin-A7 in P30 *Atf6*^+/+^ mice led to reduced expression of UPR-related genes. These findings suggest that ATF6 inhibition directly suppresses ER stress pathways that support pathological angiogenesis, particularly under hypoxic conditions. This mechanism aligns with evidence from oncology, where preclinical studies have demonstrated the critical roles of PERK and IRE1 in promoting tumor angiogenesis^16^. These insights have driven early-phase clinical trials (https://clinicaltrials.gov) exploring anti-IRE1 (NCT03950570) and anti-PERK (NCT04834778) compounds as potential cancer therapies^86,87^. The observed reduction in UPR-related gene expression may reflect a disruption of pro-angiogenic signaling and a dampened ER stress response in disease conditions, contributing to the observed reduction in neovascularization and preservation of retinal function.

Our study shows that genetic ATF6 inhibition preserves visual function and reduces neovascularization, but further research is needed to evaluate its therapeutic potential. Key steps include evaluating the ocular safety and potency of compounds that inhibit ATF6 or UPR and optimizing their delivery to the retina. If ATF6-targeting approaches prove effective in preserving vision and reducing neovascularization, they could lead to novel treatments for blinding retinal diseases.

## Materials and methods

### Animals

*Atf6*-knockout (*Atf6*^*−/−*^) mice were originally generated by the Kaufman laboratory^35^ and provided directly by Dr. Randal J. Kaufman. We have maintained and bred this line in our laboratory and used it in multiple previously published studies^36–38^. Wildtype (*Atf6*^*+/+*^) and *Atf6*^*−/−*^ mice on a pure C57BL/6J (B6J) background were used for the experiments. Both male and female animals were studied at postnatal (P) days 3, 7, 10, 12, 14, 17, 20, and 30. All animals were maintained in a specific pathogen-free (SPF) facility with unrestricted access to food and water and maintained under a 12-hour light/dark cycle. Mice were obtained from at least three different litters derived from separate breeding pairs to minimize litter-specific effects. Within each genotype, pups were randomly selected and assigned to experimental or control groups to ensure balanced representation across litters. Inclusion criteria consisted of genotyped *Atf6*^*+/+*^ and *Atf6*^*−/−*^ mice of both sexes on a C57BL/6J background with no baseline ocular abnormalities. Mice exhibiting developmental defects or signs of systemic illness were excluded prior to experimental allocation. Investigators responsible for outcome assessments and data analysis were blinded to both genotype and treatment group to reduce potential bias. Mouse husbandry and experimental procedures were approved by and conducted strictly according to the relevant guidelines and regulations of the Institutional Animal Care and Use Committee at Stanford University, Veterans Affairs Palo Alto Health Care System (Palo Alto, CA), and EyeCRO (Oklahoma City, OK) in compliance with the Association for Research in Vision and Ophthalmology Statement for the Use of Animals in Ophthalmic and Vision Research and the ARRIVE (Animal Research: Reporting of In Vivo Experiments) guidelines.

### Oxygen-induced retinopathy

The oxygen-induced retinopathy (OIR) protocol was induced in *Atf6*^*+/+*^ and *Atf6*^*−/−*^ mice as previously described^14,44^. Briefly, at P7, mouse pups of both sexes and their nursing mothers were placed in an environment with 75% oxygen (hyperoxia) for five consecutive days. They were then returned to room air at P12. Retinas were collected at P12 (end of hyperoxia), P17 (peak of neovascularization), and P20 (regression phase of neovascularization)^5,14,44^.

### Administration of Ceapin-A7 (Atf6 inhibitor)

Intravitreal injections in mice were conducted under anesthesia induced by intraperitoneal injection of ketamine (80–90 mg/kg; KETASET, Fort Dodge, IA, USA) and xylazine (5–10 mg/kg; X-Ject SA, Butler, Dublin, OH, USA). Ceapin-A7 was obtained from Sigma-Aldrich (St. Louis, MO, United States) and prepared at a concentration of 10 µM dissolved in sterile-filtered saline containing 0.01% dimethyl sulfoxide (DMSO). A volume of 1 µL of Ceapin-A7 was administered via intravitreal injection using a Hamilton syringe with a 33-gauge needle (Sigma-Aldrich Corp.). For the Ceapin-A7-treated group, both eyes of each animal were intravitreally injected with Ceapin-A7 at P30. In the control group, both eyes of each animal were intravitreally injected with 1 µL of saline containing 0.05% DMSO at P30. After injection, veterinary ophthalmic antibacterial ointment was administered to prevent infection and corneal drying. Retinas were collected 24 h post-injection for RNA-Seq analysis.

### Retina tissue preparation

Mice were anesthetized via intraperitoneal injection of ketamine (80–90 mg/kg; KETASET, Fort Dodge, IA, USA) and xylazine (5–10 mg/kg; X-Ject SA, Butler, Dublin, OH, USA). Retinal tissue was collected from the enucleated eyes at postnatal days P3, P7, P10, P12, P14, P17, P20, and P30. Following enucleation, the animals were euthanized by administering an overdose of Euthasol (200 mg/kg, Virbac Corporation, Fort Worth, TX, USA), with cervical dislocation performed as a secondary method. The cornea and lens were removed, and eyecups were fixed in 4% paraformaldehyde prepared in 0.1 M phosphate buffer (PB) for 60 min at 4°C. Post-fixation, the eyecups were transferred to 30% sucrose solution overnight at 4°C. Subsequently, the eyecups were embedded in Optimal Cutting Temperature (OCT) medium (Tissue-Tek, Elkhart, IN, USA), frozen using liquid nitrogen, and sectioned along the vertical meridian using a Leica cryostat (Leica Biosystems Inc, Buffalo Grove, IL, USA) to a thickness of 15 μm. The 15-µm-thick retinal sections were collected and mounted on Superfrost Plus slides (Fisher Scientific). For wholemount retinal preparation, retinas were carefully dissected from fixed eyecups. The ciliary body was retained during dissection to ensure the entire peripheral retina was preserved for complete area analysis. Retinas were then flattened for staining.

### Immunohistochemistry

For immunohistochemistry, 15-µm-thick cryostat sections were incubated in normal donkey serum (NDS) (Jackson ImmunoResearch Laboratories, Inc., West Grove, PA, USA) for 1 h at room temperature. Following this, the sections were incubated overnight at 4°C with primary antibodies: Alexa Fluor™ 568 conjugate Isolectin GS-IB4 (Thermo Fisher Scientific, Waltham, MA, #121412, dilution 1:1000), Alexa Fluor™ 594 conjugate Isolectin GS-IB4 (Thermo Fisher Scientific, Waltham, MA, #121413, dilution 1:1000), rabbit polyclonal antibody against ERG (Abcam, Cambridge, UK, ab92513, dilution 1:200), and Alexa Fluor™ 488 conjugate Ki-67 (Thermo Fisher Scientific, Waltham, MA, #53-5698-82, dilution 1:1000). After primary antibody incubation, sections were either washed with 0.1 M phosphate buffer (PB) (for those with conjugated antibodies) or incubated for 2 h at room temperature with Cy3-conjugated donkey anti-rabbit IgG (Jackson ImmunoResearch Laboratories, dilution 1:500). For triple-label studies, sections were incubated overnight with a mixture of primary antibodies followed by a 2-hour incubation with the secondary antibody. The sections were then washed for 30 min with 0.1 M PB, cover-slipped with VECTASHIELD HardSet™ Antifade Mounting Medium with DAPI (Vector Laboratories, Newark, CA, #H-1000-10), and analyzed using a Nikon A1R confocal microscope (Nikon, NY, USA). Immunofluorescence images were processed using Nikon NIS Elements and ImageJ (National Institutes of Health, imagej.net). The brightness and contrast of the images were adjusted using Adobe Photoshop 7.0 (Adobe Systems, Inc., San Jose, CA, USA). Wholemount immunostaining was conducted in the same manner except that the incubation times with the primary antibodies were extended to 2 nights.

### RNA sequencing

RNA sequencing was carried out as described in previous studies^55,88^. A sample size of five whole retinas each from P17 *Atf6*^*+/+*^ OIR, P17 *Atf6*^*−/−*^ OIR, P30 wildtype saline-treated, and P30 wildtype Ceapin-A7-treated mice were collected, and RNA was extracted according to the manufacturer’s instructions. BGI’s Eukaryotic Strand-specific Transcriptome Resequencing service (http://biosys.bgi.com) performed the RNA sequencing, utilizing the DNBSEQ stranded mRNA library to produce paired-end 100 bp reads, achieving 30 million reads per sample. The sequencing reads were aligned to the Mus_musculus_GCF_000001635.27_GRCm39 genome using HISAT2 alignment software (v2.0.4). Gene expression levels and normalized reads in FPKM were quantified using RSEM software (v1.2.18)^89^. Differential expression analysis between *Atf6*^+/+^ and *Atf6*^−/−^ OIR retinas, as well as between saline-treated and Ceapin-A7-treated retinas, was conducted using the DESeq2 package (v1.4.5)^90^.

### Functional enrichment analysis

Functional enrichment analysis was performed using g:Profiler (https://biit.cs.ut.ee/gprofiler/), focusing on Gene Ontology (GO) terms in the Biological Process category. Input genes were selected from RNA-Seq data based on significant differential expression between *Atf6*^*+/+*^ and *Atf6*^*−/−*^ OIR retinas (log_2_fold change, p *≤* 0.05, expression > 0.1 FPKM), resulting in 2,096 differentially expressed genes (DEGs) from an initial pool of 15,524. As an alternative approach, Gene Set Enrichment Analysis (GSEA) software (https://www.broadinstitute.org/gsea/) was also used. Pre-ranked gene lists, ordered by expression relative to wild-type controls, were analyzed using the GO reference database with weighted scoring. GSEA enrichment plots were generated to visualize pathway-level changes.

### Electroretinogram

Full-field electroretinogram (ERG) analyses were conducted using an Espion system (Diagnosys, Lowell, MA, USA). Following a minimum of 12 h of dark adaptation, the animals were anesthetized with an intraperitoneal injection of ketamine (80–90 mg/kg; KETASET, Fort Dodge, IA, USA) and xylazine (5–10 mg/kg; X-Ject SA, Butler, Dublin, OH, USA). Under dim red light, Proparacaine HCL Ophthalmic Solution (0.5%; Bausch and Lomb, Bridgewater, NJ, USA) was applied as a local anesthetic to the pupils. Subsequently, Tropicamide Ophthalmic (1%; Bausch and Lomb, Bridgewater, NJ, USA) and Phenylephrine HCL Ophthalmic Solution (10%; AKORN, Lake Forest, IL, USA) were administered as a cycloplegic agent and a dilator, respectively. For scotopic response assessment, the dark-adapted, dilated eyes were exposed to a stimulus intensity of 40 (S) cd.s/m². To evaluate the photopic response, the animals were light-adapted for 7 min before a strobe flash with an intensity of 10 (S) cd.s/m² was presented to the dilated eyes. Fifteen repeated flashes were averaged to obtain the final waveform, with the photopic b-wave amplitude measured from the a-wave trough to the b-wave peak.

### Quantification of retinal vasculature in OIR

Vaso-obliteration was assessed by outlining the central avascular area and total retinal area using the Polygonal Lasso tool in Photoshop (Adobe Systems, San Jose, CA) and expressed as the central avascular area in relation to the total retinal area (% avascular area) at P12, P17, and P20. For neovascularization, individual neovascular tufts and clusters were outlined with a white dotted line using the same software at P17 and P20, and the total neovascular area was expressed in relation to the total retinal area (% neovascular area). Wholemount images were imported into NIH’s ImageJ software (National Institutes of Health, Bethesda, MD; available at http://rsb.info.nih.gov/ij/index.html) for quantification and retinal area measurement^5,14^.

To quantify proliferating endothelial cells in the *Atf6*^+/+^ and *Atf6*^−/−^ OIR mice, retinas were examined to compare Ki-67-immunoreactive cells (a marker for proliferating cells)^91^ in ERG-positive cells (an endothelial cell marker)^92,93^ within IB4-immunoreactive blood vessels. Two regions, each located 500 μm away from the optic disc, were selected from each retina for measurement. At these locations, serial optical sections were imaged by confocal microscopy. Every Ki-67-immunoreactive cell within the ERG-immunoreactive region of the blood vessels was counted by assessing its colocalization with IB4 immunoreactivity.

### Quantification of retinal vasculature in early development

The retinal areas of wholemount retinas from *Atf6*^+/+^ and *Atf6*^−/−^ mice were measured at P3, P7, P10, and P14 using ImageJ software (National Institutes of Health, Bethesda, MD). To evaluate the vascular fraction, the vascular area and the total retinal area were outlined using the Polygonal Lasso tool in Photoshop (Adobe Systems, San Jose, CA), and the vascular area was expressed as a ratio to the total retinal area at P3, P7, P10, and P14.

High-resolution images of retinal wholemounts stained with IB4 were used to analyze the number of vascular branches. Three fields of 175 μm × 175 μm were sampled from the central (250 μm away from the optic disc), mid-peripheral (750 μm away from the optic disc), and peripheral (1.25 mm away from the optic disc) areas in the superior region of wholemount retinas. Using ImageJ, images of these areas were thresholded to create a binarized image in which the vascular area is positive and the background is negative. Binarized images were then processed into skeletons using the Skeletonize plugin, and branch numbers were quantified using the AnalyzeSkeleton plugin^94^. Endothelial tip cells were quantified along the leading edge of the developing vascular plexus using ImageJ as described in a previous study^63^.

### Measurement of outer nuclear layer (ONL) thickness

The thickness of the ONL was measured at 100 μm intervals, beginning 500 μm away from the optic disc. For each retinal section, five measurements of ONL thickness were taken, spaced approximately 100 μm apart, and averaged. Layer thickness measurements were collected from three retinas each from *Atf6*^+/+^ and *Atf6*^−/−^ mice.

### Statistical analysis

All data are expressed as mean ± standard error of the mean (SEM). A p-value of less than 0.05 was considered significant and annotated as follows: *p < 0.05, **p < 0.01, ***p < 0.001, and ****p < 0.0001. Student’s t-test or Welch’s t-test was used to compare two group means, depending on variance equality, which was assessed by an F-test. Two-way ANOVA followed by Tukey’s multiple comparisons test was used to evaluate the effects of genotype (*Atf6*^+/+^ vs. *Atf6*^−/−^) and developmental timepoint (e.g., P12 vs. P17 or P17 vs. P20). Violin plots were generated using the log_2_(fold change) data from the RNA-Seq differential expression analysis. Differences in the expression of gene sets were evaluated for statistical significance using the Two-Tailed Wilcoxon Signed Rank Test. All statistical analyses were performed using GraphPad Prism 10 (GraphPad Software, San Diego, CA).

### Statistical power and sample size estimation

Post hoc power analyses were performed using GraphPad Prism 10 to evaluate whether the sample sizes used in the experiments provided sufficient statistical power. For each comparison, group means, 95% confidence intervals (CIs), and standardized effect sizes (Cohen’s d) were calculated. Sample size estimates required to achieve 80% power were based on unpaired t-tests (two-tailed, α = 0.05). Analyses were conducted for neovascularization and vaso-obliteration. Detailed results of these analyses are reported in the Results section.

## Supplementary Information

Below is the link to the electronic supplementary material.

Supplementary Material 1

Supplementary Material 2

Supplementary Material 3

Supplementary Material 4

Supplementary Material 5

Supplementary Material 6

Supplementary Material 7

Supplementary Material 8

## Data Availability

RNA-Seq data can be accessed at the NCBI Gene Expression Omnibus (GSE292097). All additional data are included in the article and supporting information.
