# Supplementary material for: Targeting ATF6 reduces pathological neovascularization and improves visual outcomes in retinal disease models
Source: Sci Rep. 2025 Sep 26;15:33070. doi: 10.1038/s41598-025-15393-y (PMC12475287; doi:10.1038/s41598-025-15393-y)
Supplement: Supplementary file 8 — Supplementary Material 8 [file 41598_2025_15393_MOESM8_ESM.pdf]

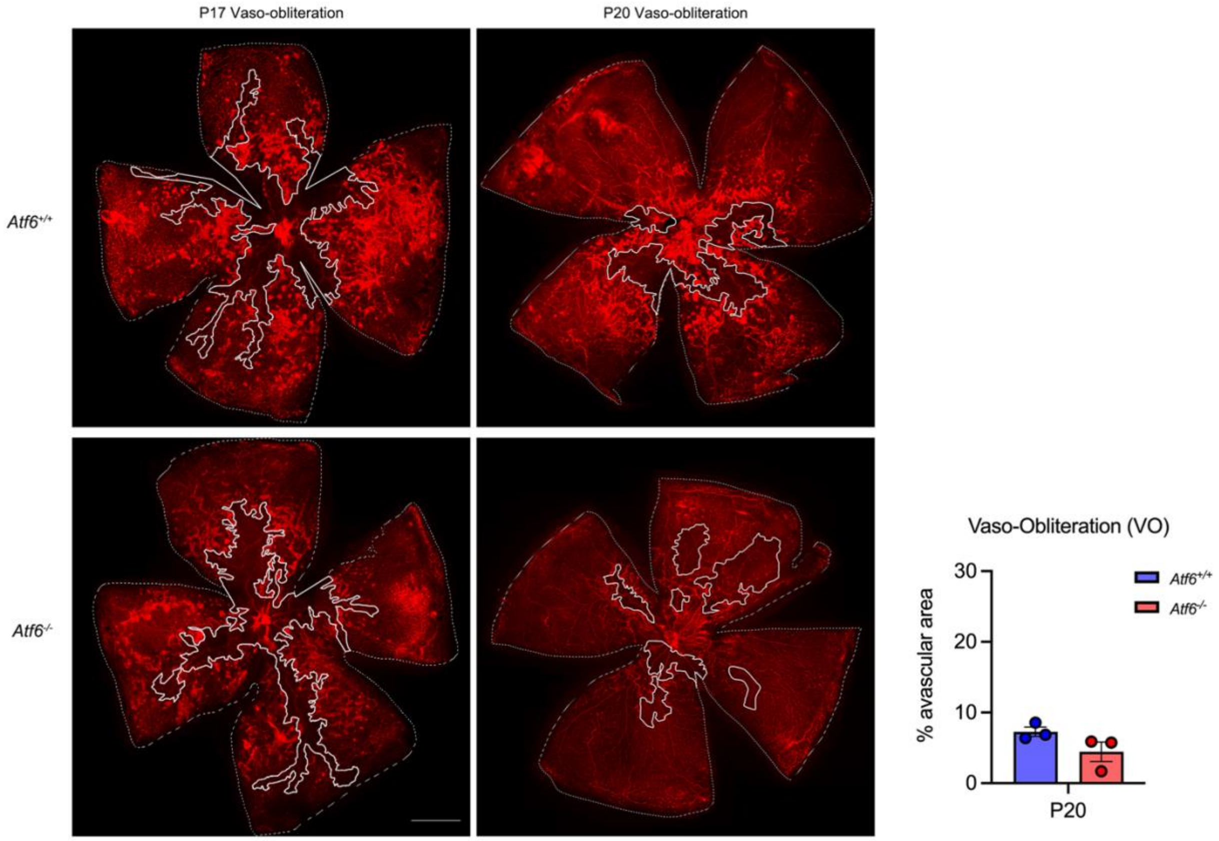

Supplemental Figure 1. Representative flat-mounted retinal images showing avascular (vaso-obliterated) areas at P17 (left) and P20 (right) in *Atf6*$^{+/+}$ and *Atf6*$^{-/-}$ OIR retinas. At P17, both genotypes display central avascular zones (solid white outline). By P20, avascular areas are minimal in both groups. The quantification graph shows low VO at P20 in both genotypes (*Atf6*$^{+/+}$ = 7.28%, *Atf6*$^{-/-}$ = 4.44%, n=3/group), with no statistically significant difference (Student's t-test). Total retinal area is outlined in each panel. Scale bar, 500 µm.

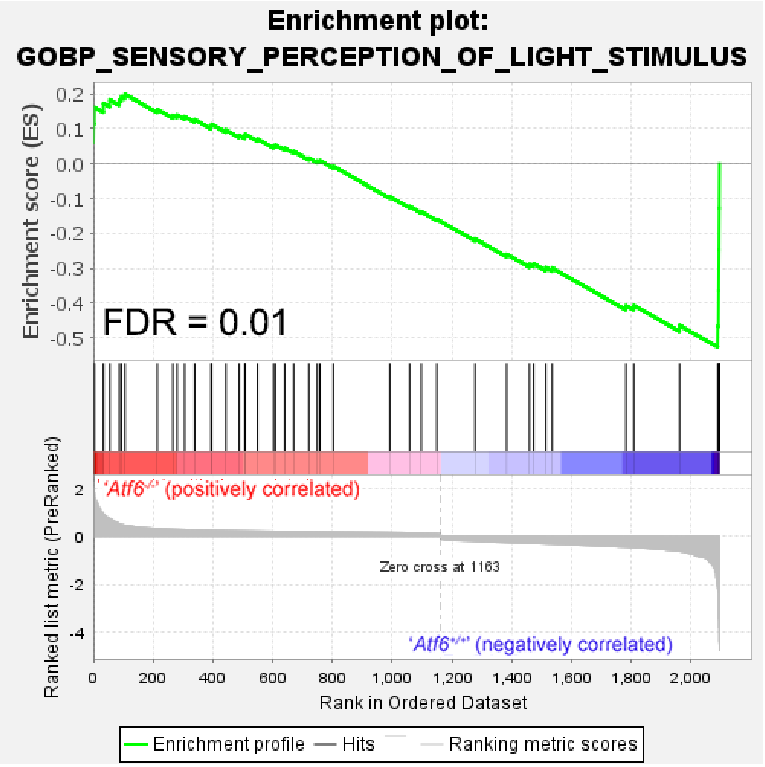

Supplemental Figure 2. Gene Set Enrichment Analysis (GSEA) plot for the Gene Ontology term "sensory perception of light stimulus" comparing *Atf6*$^{+/+}$ and *Atf6*$^{-/-}$ OIR retina. The enrichment score (ES) curve is shown in green (top), with a peak indicating the point of maximum enrichment. The middle panel displays vertical black lines representing the positions of genes from the gene set within the ranked gene list. The bottom panel shows the ranking metric score, with genes upregulated in *Atf6*$^{-/-}$ OIR retinas on the left (red, positively correlated) and downregulated genes on the right (blue, negatively correlated). False Discovery Rate (FDR) = 0.01.
